# The use of remote Femto Second technology in the preparation of black silicon material and optical devices

**DOI:** 10.1371/journal.pone.0283456

**Published:** 2023-03-27

**Authors:** Ninghui Li, Yuan Chen

**Affiliations:** College of Traffic Engineering, Nanjing Vocational University of Industry Technology, Nanjing, China; Sungkyunkwan University, REPUBLIC OF KOREA

## Abstract

The research aims to study the application of remote Femto Second (FS) technology in black silicon material preparation and optical devices. Based on the principle and characteristic research of FS technology, the interaction between FS and silicon is adopted to propose a scheme for preparing black silicon material through experiments. Moreover, the experimental parameters are optimized. Then, the scheme of using the FS for etching polymer optical power splitter as a new technical means is proposed. In addition, while ensuring processing accuracy, the appropriate process parameters of laser etching photoresist are obtained. The results show that the performance of black silicon prepared with SF_6_ as the background gas is greatly improved in the 400-2200nm range. However, the performance of black silicon samples with the two-layer structure etched at different laser energy densities has little difference. Black silicon with a Se+Si two-layer film structure has the best optical absorption performance in the infrared range of 1100nm-2200nm. Besides, the optical absorption rate is the highest when the laser scanning rate is 0.5mm/s. In the band of >1100nm, when the maximum laser energy density is 6.5kJ/m2, the overall absorption of the etched sample is the worst. The absorption rate is the best when the laser energy density is 3.9kJ/m2. It suggests that the proper parameter selection greatly impacts the quality of the final laser-etched sample.

## 1. Introduction

Femto Second (FS) has undergone 40 years of development from the initial dye laser to the sapphire laser to the latest photonics crystal fiber laser [1–3]. With technical advancement, the pulse width of FS becomes increasingly shorter, the peak power of the pulse becomes increasingly larger, and FS gradually moves from laboratory to industrial application [4–6]. Hence, it has multiple excellent fine and micro-processing features that traditional technology does not have, such as special-shaped screen cutting, micro-fine processing of brittle materials, and myopia surgery [7, 8]. With the continuous development of semiconductor materials, integrated circuits are increasingly miniaturized and complex, which requires continuous refinement of processing technology. The limitations of traditional manufacturing technology are increasingly prominent [9, 10]. For example, the thickness of the semiconductor wafer continues to thin, the size continues to shrink, and the processing difficulty is also gradually increasing [11]. Thereby, it can cut a quite thin layer of material without affecting the underlying material, thus ensuring the processing quality and accuracy of the material [12–14].

The preparation process of black silicon is quite complex. The interaction between remote FS technology and black silicon can help to complete this complex process [15–17]. However, the process of FS etching to prepare black silicon with a bush-like taper structure is particularly complex. Hence, the principle of black silicon formation is not clear at present [18]. Given this problem, multiple scholars have their own views on the research of FS technology. According to the transmission electron microscopy results, Li et al. (2018) [19] observed multiple phases in the laser irradiation layer, and prepared the bulk photodiode based on Schottky. The broadband photodiode shows good thermal stability and optical response when the reverse bias is 10V. Su et al. (2020) [20] first tried to prepare photodiodes based on femtosecond filament confrontation silicon-based fixed texture. They opened the way for rapid and large-scale manufacturing of infrared photoelectric devices. Paulus et al. (2021) [21] demonstrated how to selectively remove the harmful amorphous and polycrystalline silicon surface layer produced by FS processing through ion beam etching, while leaving the following crystal infrared absorption silicon. Liu et al. (2022) [22] pointed out that FS-textured black silicon can easily cause heavy minority carrier recombination when exposed to laser irradiation. Therefore, a high minority carrier lifetime of 2ms was obtained through the optimized wet chemical etching process without sacrificing the optical characteristics of the sample. Besides, black silicon is a new electronic material that can greatly improve photoelectric conversion efficiency and reduce energy reflection. It has broad application prospects in the field of wavelength expansion of silicon-based materials.

FS is currently the shortest pulse that humans can obtain under experimental conditions. Its accuracy is ±5μm, and it has very high instantaneous power. Its instantaneous power can reach millions of gigawatts, which is hundreds of times more than the current total power generation in the world [23]. FS can achieve accurate target focusing and positioning and focus on ultra-fine spatial areas much smaller than the diameter of the hair. Under the action of FS, substances will produce bizarre phenomena. Gaseous substances, liquid substances and solid substances will instantly become plasma. FS has a very short pulse width and can generate extremely high power in an instant. Hence, it will not generate excess heat, thus avoiding the cracking, damage, melting and other phenomena of materials produced during processing. Then, quite high-quality processing results can be obtained, which is particularly crucial for the fine processing of brittle materials such as glass and sapphire. FS is increasingly widely employed in industrial production due to its various excellent characteristics.

The main application of FS can be summarized into three aspects: in the ultra-fast field, in the ultra-strong field and in ultra-micro processing. FS plays the role of rapid process diagnosis in ultra-fast phenomenon research. It is like an extremely fine clock and an ultra-high-speed "camera", which can analyze and record some rapid processes in nature, especially at the atomic and molecular levels. Its application in the ultra-strong field is attributed to the fact that the peak power and light intensity of the FS pulse with certain energy can be extremely high. The electromagnetic field corresponding to such strong light will be much larger than the Coulomb field in the atom, so it is easy to strip all the electrons in the atom. It can be adopted to generate coherent X-rays and other extremely short wavelength light, and to research controlled nuclear fusion. FS ultra-micro processing is closely related to advanced manufacturing technology and can more directly promote the development of some key industrial production technologies.

It reveals that FS is widely applied to the fine processing of semiconductor materials, but there are still some problems. Therefore, based on the principle and characteristic research of FS technology, using the interaction between FS and silicon, this research puts forward a scheme of preparing black silicon material through experiments and optimizes the experimental parameters. The research’s originality is to propose a new technique of etching polymer optical power splitter with FS. This method ensures processing accuracy and obtains the appropriate process parameters for laser etching photoresists. Compared with the research methods proposed in other literature reviews, the method proposed here has crucial reference value for the development of FS etching.

## 2. Research method

### 2.1 Preparation of black silicon material based on remote FS technology

In order to analyze the optical properties of black silicon, it is essential to quantitatively compare the preparation methods and numerical models of black silicon. In the photo-generated carrier circuit less than 1.1μm, the electricity generated by infrared sunlight needs to be extracted and measured by black silicon material. Moreover, black silicon materials can introduce impurity levels through supersaturation doping, thus expanding the response range of black silicon devices. It can well make up for the shortcomings of the narrow response range and low quantum efficiency of traditional silicon-based detectors. Compared with ordinary laser, the pulse width of FS can reach 10^−15^ seconds, and the peak power after amplification can reach 1021 W/cm^2^. However, the preparation of black silicon by FS is to generate a series of microstructures on the surface of silicon by high-power laser scanning. First, the changes occurring when the single pulse FS is radiated to the silicon surface are analyzed. When the silicon surface absorbs the single pulse energy, the interaction between the laser energy and the silicon will create a plasma layer with a thickness of about 10μm on the surface layer. After the relaxation process, the energy is transferred to the lattice, causing the lattice to vibrate, and the heat generated by the vibration will melt the film. This process occurs between two FS pulses and is defined as the initial state. The temperature distribution at this time is recorded as follows:

$$T(r,z,t=0)=T_{0}exp(-\alpha z)exp\left(-\frac{r^{2}}{r_{0}^{2}}\right)$$ (1)

**Fig 1:**
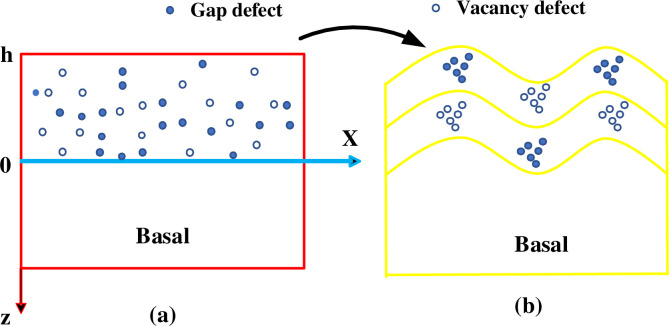
State model diagram of black silicon (a. initial state; b. model diagram after multi-pulse action).

*T*_0_ represents the temperature of the facula center on the *Z* = 0 plane, and *α* is the base optical absorption coefficient. Fig 1 shows the definition of the coordinate axis.

In Fig 1, the silicon substrate irradiated by a single-pulse laser mainly undergoes molten and melting. It is assumed that the thickness of the melting layer and molten layer is *l*_*a*_ and *l*_*m*_, respectively. According to the information provided in the literature [24], it can be obtained that:

$$l_{a}=\alpha^{-1}ln\frac{T_{0}}{T_{a}}$$ (2)

$$l_{m}=a^{-1}ln\frac{T_{0}}{T_{m}}$$ (3)

*T*_*a*_ is the temperature at the time of melting, and *T*_*m*_ represents the molten point of the molten layer. After the single pulse energy is absorbed, the part used for the surface melting layer I_a_ is:

$$W_{a}=W\left(I-\frac{T_{a}}{T_{0}}\right)$$ (4)

The part used for the molten layer I_m_−I_a_ at the bottom is:

$$W_{m}=W-W_{a}=W\frac{T_{a}}{T_{0}}$$ (5)

Then, the two types of melting can be described as follows.

When the energy is low,

$$T_{0}∼T_{a},W_{a}\ll W∼W_{m}$$ (6)

Most of the absorbed energy is used for the molten process. Then,

$$\left(T_{0}∼T_{a},T_{a}\gg T_{m}\right)$$ (7)

There is:

$$\frac{l_{a}}{l_{m}}=\frac{ln(T_{0}/T_{a})}{ln(T_{0}/T_{m})}\approx \frac{ln(T_{0}/T_{a})}{ln(T_{a}/T_{m})}\ll 1$$ (8)

For the initial molten layer *l*_*m*_, the molten layer has only one layer thickness, which can be ignored. This type of melting process is liquid phase melting. When the energy is high,

$$T_{0}\gg T_{a},W_{a}∼W\gg W_{m}$$ (9)

At this time, most of the absorbed energy is used for the melting process. Similarly, in the case of high energy, there are:

$$\left(T_{0}\gg T_{a}\gg T_{m}\right)$$ (10)

$$\frac{l_{a}}{l_{m}}=\frac{lnT_{0}-lnT_{a}}{lnT_{0}-lnT_{m}}∼1$$ (11)

For the initial molten layer *l*_*m*_, all the molten layers have been evaporated, and this melting process is called solid phase melting.

**Fig 2:**
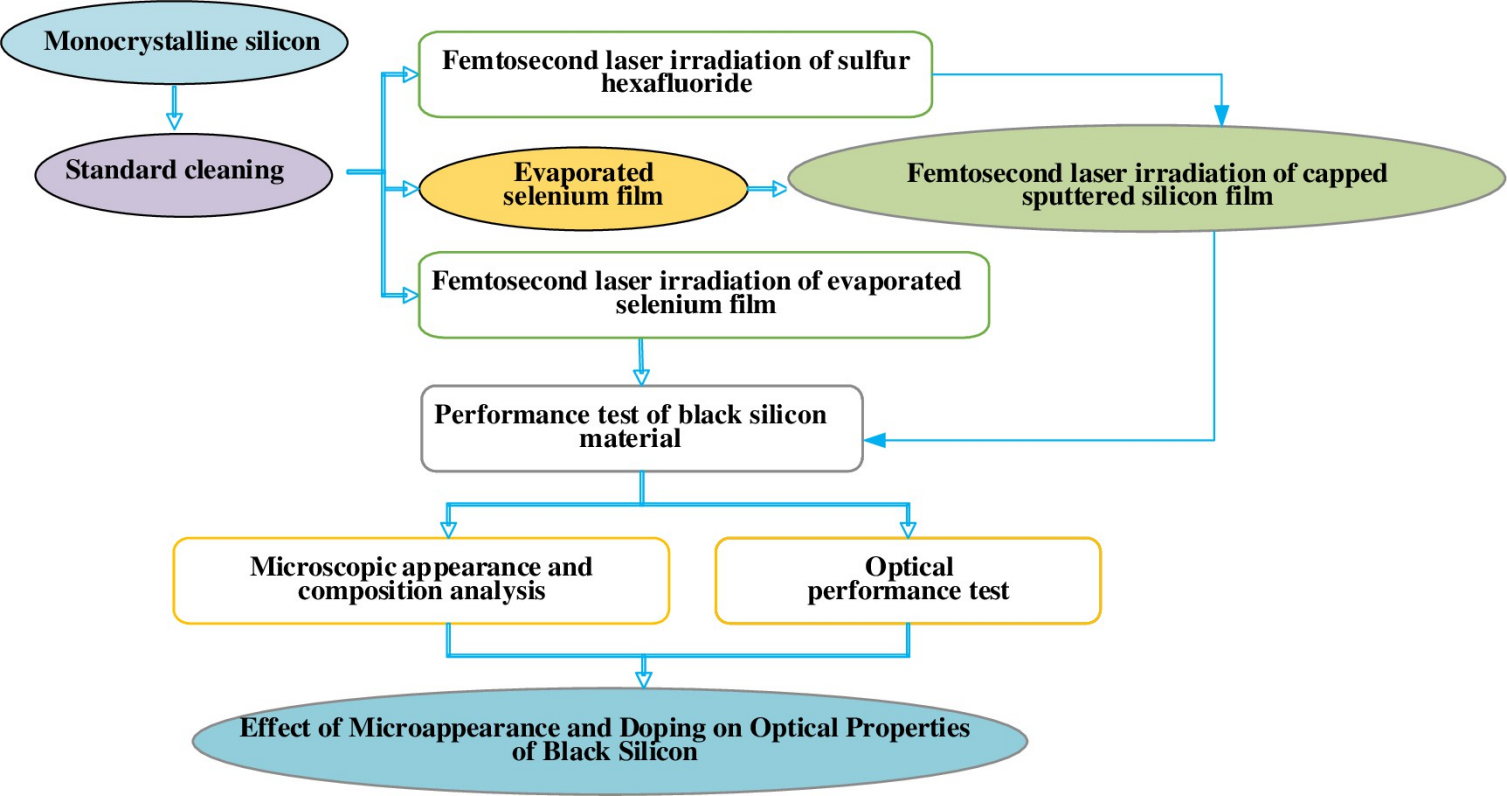
Flow chart of processing doped black silicon material by FS irradiation method.

**Fig 3:**
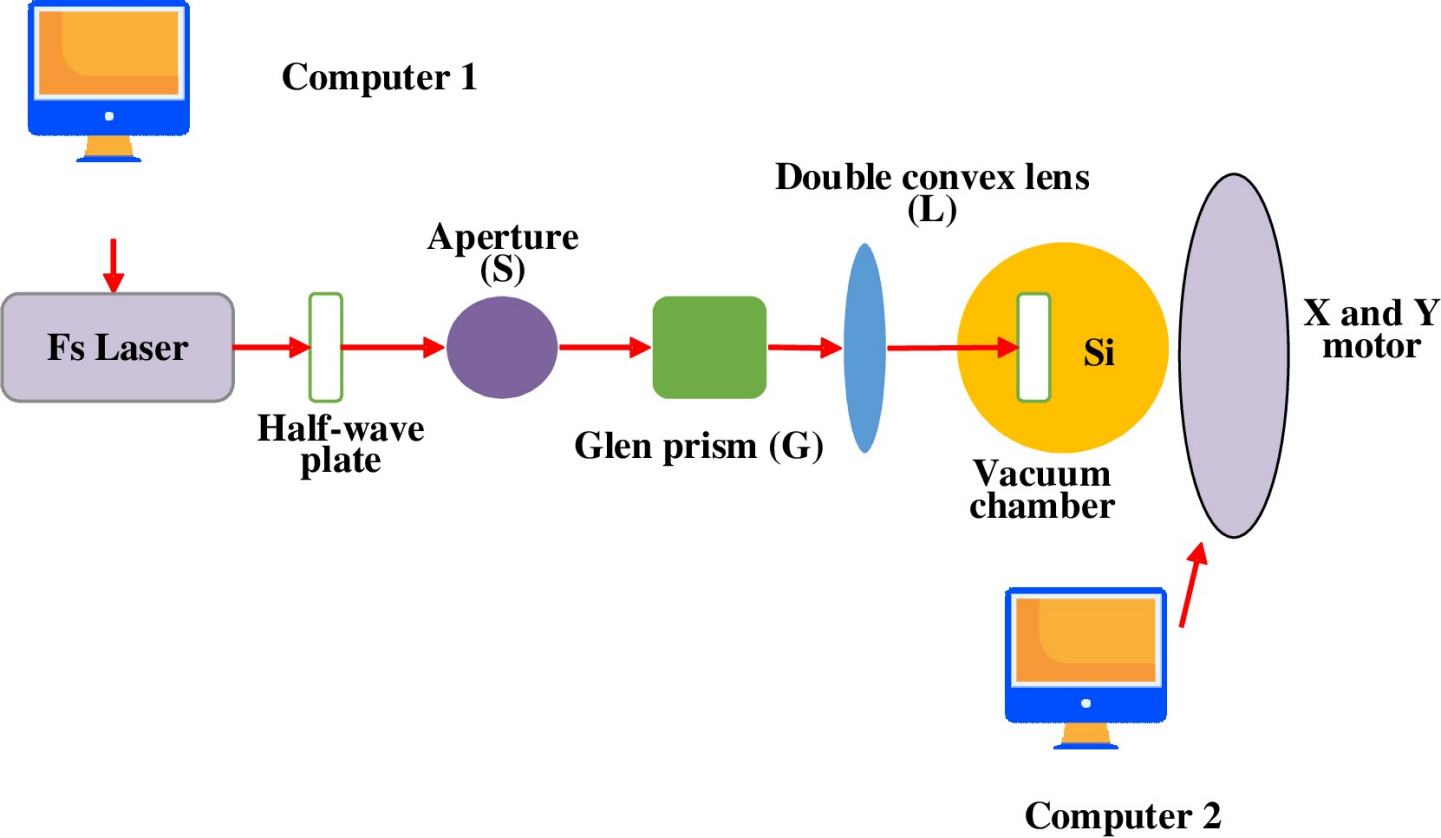
Schematic diagram of FS-based black silicon processing optical system.

After the action of FS and silicon, the black silicon material can be prepared. Fig 2 presents the specific process:

Here, an FS device is selected as the experimental equipment for the preparation process of black silicon material, and the model is MaiTai 100fs. During processing, the central wavelength of the laser output is 800nm. At this wavelength, the output power does not exceed 5W, and the output mode is TEM_00_ mode. The initial laser is linearly polarized light, and the laser beam is Gaussian. Fig 3 presents a schematic diagram of the optical path of the processing experiment.

**Fig 4:**
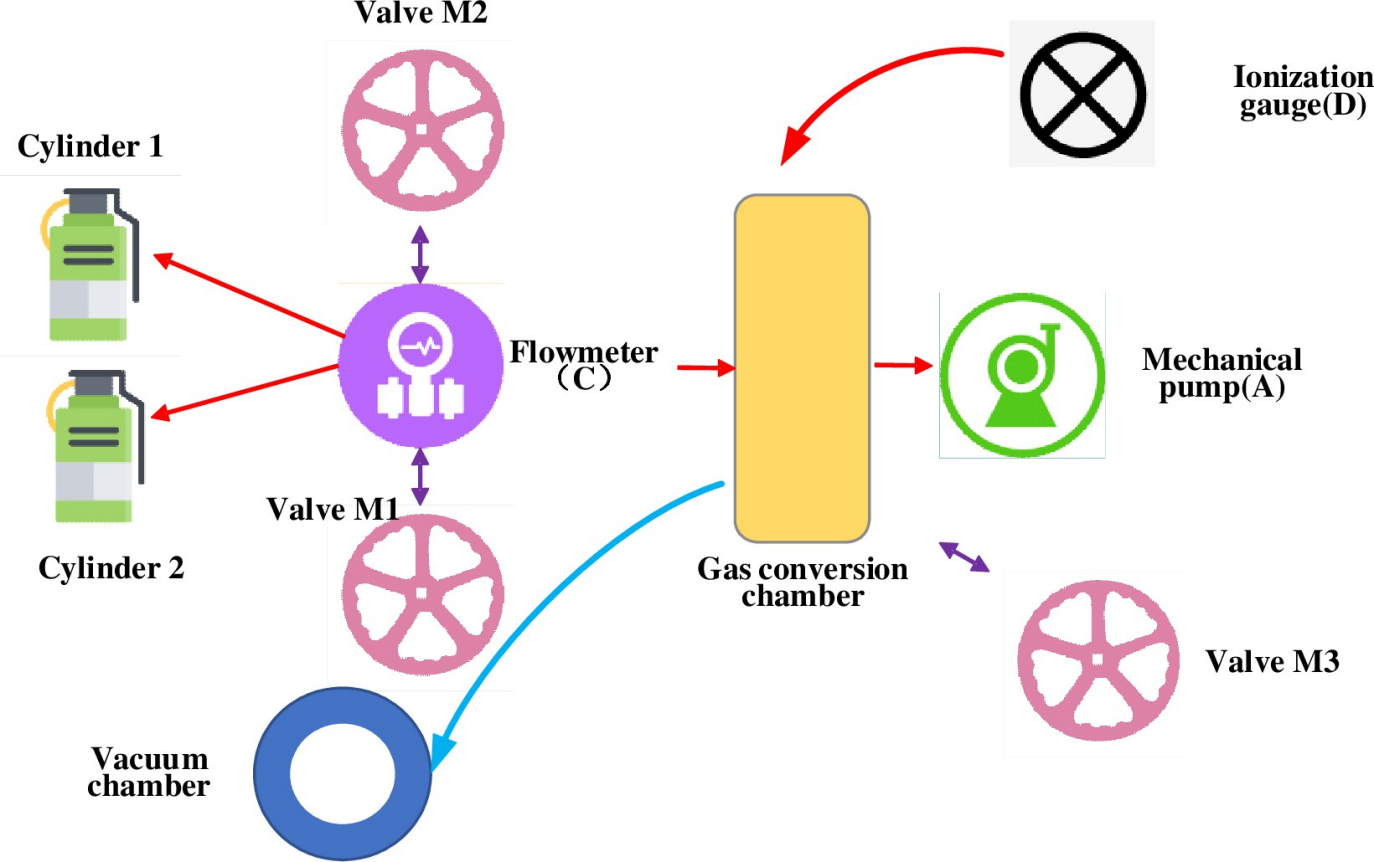
A schematic diagram of FS-based black silicon processing vacuum system.

Fig 3 suggests that after FS collimation, it first passes through a half-wave plate. Then, it passes through an aperture (S) and a Glan prism (G) to adjust the incident laser’s polarization direction, spot size, power and other parameters. Then, the laser speed is focused through a biconvex lens with a focal length of 10cm. Finally, the laser beam passes through the quartz glass of the vacuum chamber and is perpendicular to the silicon surface. Then, the X-Y translation table and computer 2 are connected through a serial line. Fig 4 is a schematic diagram of the vacuum system used in the experiment.

Fig 4 suggests that the sample is fixed at the bottom of the vacuum chamber of the vacuum system. The vacuum chamber is connected with gas conversion chamber B through a long stainless steel corrugated pipe. The longer corrugated pipe can ensure the vacuum chamber has sufficient space for movement during the black silicon processing. Then, the gas conversion chamber B is connected with the mechanical pump A by another corrugated pipe, and the conduction and sealing between the two are controlled by valve M3. A mechanical pump can make this system obtain a 10^-2^Pa vacuum. The ionization vacuum gauge on the gas conversion chamber B is adopted to measure the vacuum degree of the whole system.

The silicon wafer used in this experiment’s preparation of black silicon is cut from a four-inch single-sided polished silicon wafer. There are two types of silicon wafers, with a size of 10mm * 10mm. Table 1 is the basic specifications.

**Table 1:** Basic specifications of single crystal low resistance (high resistance) N-type silicon.

Type		Parameter
Crystal type		single crystal
Doping		Phosphorus (P)
Surface orientation		<100>
Resistivity (Ω˙cm)	Low-resistance N-type silicon	8~12
	High-resistance N-type silicon	≥2000
Thickness (μm)		500±25

The specific processing experiment process is as follows.

Step 1: open the vacuum chamber of the abovementioned vacuum system and wash the quartz glass and chamber with deionized water. Then, it is essential to wipe with a small amount of acetone dipped in a dust-free cloth, and finally dry with a dry dust-free cloth. The prepared sample is glued to the inner wall of the chamber with double-sided adhesive, and the vacuum chamber is closed and fixed on the X-Y mobile table.

Step 2: it is to start the FS.

Step 3: it is to start the mechanical pump. When the ionization gauge pointer reaches the minimum value, the mechanical pump will work longer for a while. The valve and the mechanical pump will be closed after it is estimated that the air pressure in the chamber is pumped to 10^-2^Pa.

Step 4: it is to open the valve of gas cylinder 1 or 2 and open one of the valves of the floating value flowmeter. Next, the SF_6_ gas required in this experiment needs to be injected into the vacuum chamber, and the flowmeter valve should be closed after reaching the required air pressure.

Step 5: it is the sample etching. Computer 2 is adopted to shift the upper right corner of the sample in the vacuum chamber to the focus of the FS pulse. Then, the Δx and y values are set in the calculation, and the time in the program is changed to the time in this experiment. Then, automatic scanning is conducted.

Step 6: the black silicon sample in the culture dish is put into the acetone beaker to clean the residue.

Step 7: the sample is rinsed with deionized water and then dried.

The whole process uses low-frequency ultrasound to reduce the damage to the surface structure of the sample caused by ultrasound.

### 2.2 Application of FS technology in optical devices

The micromachining technology of FS has received great attention since its formation. In addition to its application in black silicon preparation, it is also applied in optical devices. It is a new technology for processing polymer optical waveguides. The processing process is affected by various factors, such as pulse width, spot diameter, and scanning speed. The calculation method of light spot diameter is:

$$D=\frac{4fM^{2}\lambda }{\pi d_{L}}$$ (12)

*f* is the focal length of the objective lens, *M* is the light beam quality factor, *λ* is the wavelength, and *d*_*L*_ is the spot diameter before focusing.

With the emergence of 5G technology, people’s demand for information capacity and transmission speed has peaked. The transmission delay and loss of traditional on-chip interconnection can no longer meet the requirements. The main photonic devices have received the attention of researchers, such as optical waveguide splitters and optical switches. To solve this problem, combining optoelectronic technology, replacing electrical interconnection with optical interconnection and combining micro-nano electronic technology can greatly improve the transmission efficiency of signals between chips. The lithography technology used in preparing the optical power splitter is the main technology for processing polymer optical waveguides. Its processing steps are quite complicated, and the processing accuracy is difficult to control. Thereby, FS micromachining technology is adopted to solve this problem. The information provided in the literature is analyzed [25]. The optical power splitter is prepared by FS technology, and the physical properties of the photoresist are analyzed. Table 2 shows the physical specifications of the photoresist.

**Table 2:** Physical specifications of photoresist.

Type	Parameters
Index of refraction	1.566
Thermal stability	315
Thermal conductivity	0.3
Coefficient of thermal expansion	52
Young’s modulus	2.0
Permittivity	3.2

The specific steps of preparation are as follows.

1. Glass substrate cleaning. A glass substrate with the size of 20x20mm is taken out and soaked in a beaker containing 95% ethanol solution. Then, the beaker is placed in the ultrasonic cleaner. After about 20 minutes of cleaning, the glass substrate is taken out. The ethanol on the surface of the glass substrate is blown dry with a nitrogen nozzle to make the surface of the glass substrate dry and clean.
2. Spin-on PR Coating. The Spin-on PR Coating process is to spread the liquid photoresist coated on the glass substrate through the centrifugal force generated by the homogenizer’s rotation speed. The purpose is to control the thickness of the photoresist. Here, the three-speed glue mix method is adopted: low-speed spin coating, medium-speed glue spread, and high-speed glue spread.
3. Bake. The sample is heated and solidified by high-temperature baking. The glass substrate obtained in the previous step is put into the vacuum drying oven. A high-power vacuum pump is employed to reduce the pressure in the oven to -0.1Mpa. The drying oven is employed to heat it to 120°C. Then, the sample is taken out after baking.
4. FS etching. The baked sample is placed on the clamping device of the workbench. The power is set to 100mW, the speed is set to 10mm/s, and the defocusing amount is set to 0μm. The line distance of the machining path is 5μm. An automatic etching is performed according to the generated path instructions.

## 3. Results and discussion

### 3.1 Morphological changes of black silicon during the experiment

**Fig 5:**
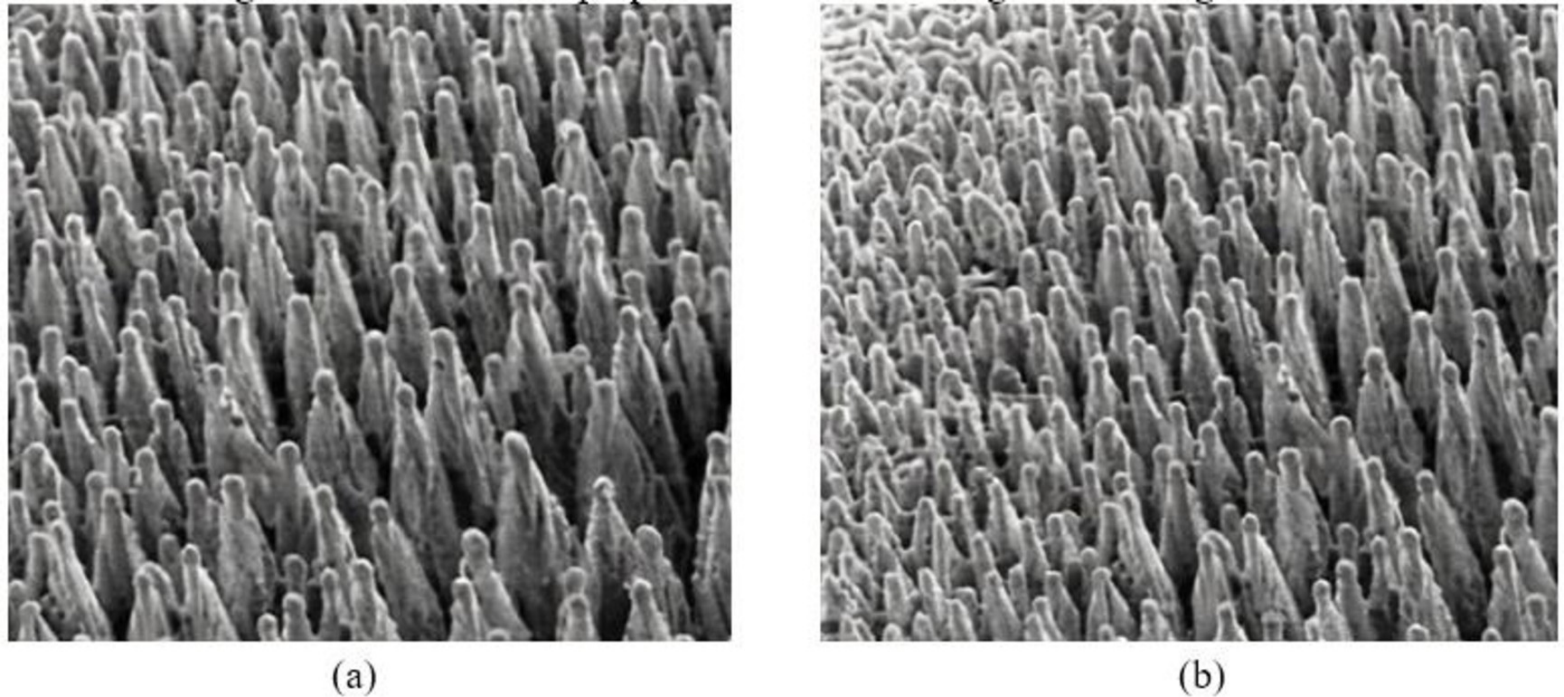
Scanning electron microscope diagram of FS-based black silicon (a. background gas is SF_6_; b. background gas is N_6_).

The FS device and optical path system mentioned above are employed. The background gas is N_6_ and SF_6_, respectively, the laser scanning speed is 0.5mm/s, and the gas pressure is about 0.09Mpa. Fig 5 shows a schematic diagram of black silicon prepared under these two gases at a magnification of 5000 times. The surface of the silicon wafer is constructed through FS. The reading scale is as follows. The laser beam adopts a lens with a focal length of 280mm. The size of the image scale area is 10mm * 10mm.

Fig 5 suggests that different cones are arranged irregularly, and the size of the cone tip is also inconsistent. The black silicon cone made with SF_6_ as the background gas has a relatively uniform volume, neat arrangement, and sharp cone tip. However, the black silicon cone made with N_6_ as the background gas is relatively large in volume and its tip is not very sharp. Moreover, the formation of surface spikes is also affected by the wavelength of light, pulse width and gas type, and the light absorption of black silicon is different under different background gases.

### 3.2 Changes in optical properties of black silicon during the experiment

**Fig 6:**
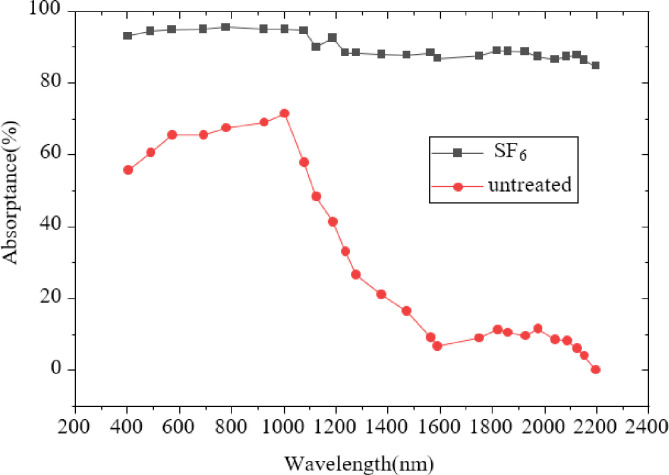
Comparison of absorption spectra of black silicon sample and monocrystalline silicon etched with SF_6_ as background gas.

In order to verify the reliability of the spectrum tester, the standard silicon absorption spectrum recognized in the industry is used as the standard for comparison. The absorption spectrum of the untreated silicon sample is tested. Fig 6 presents the comparison results.

**Fig 7:**
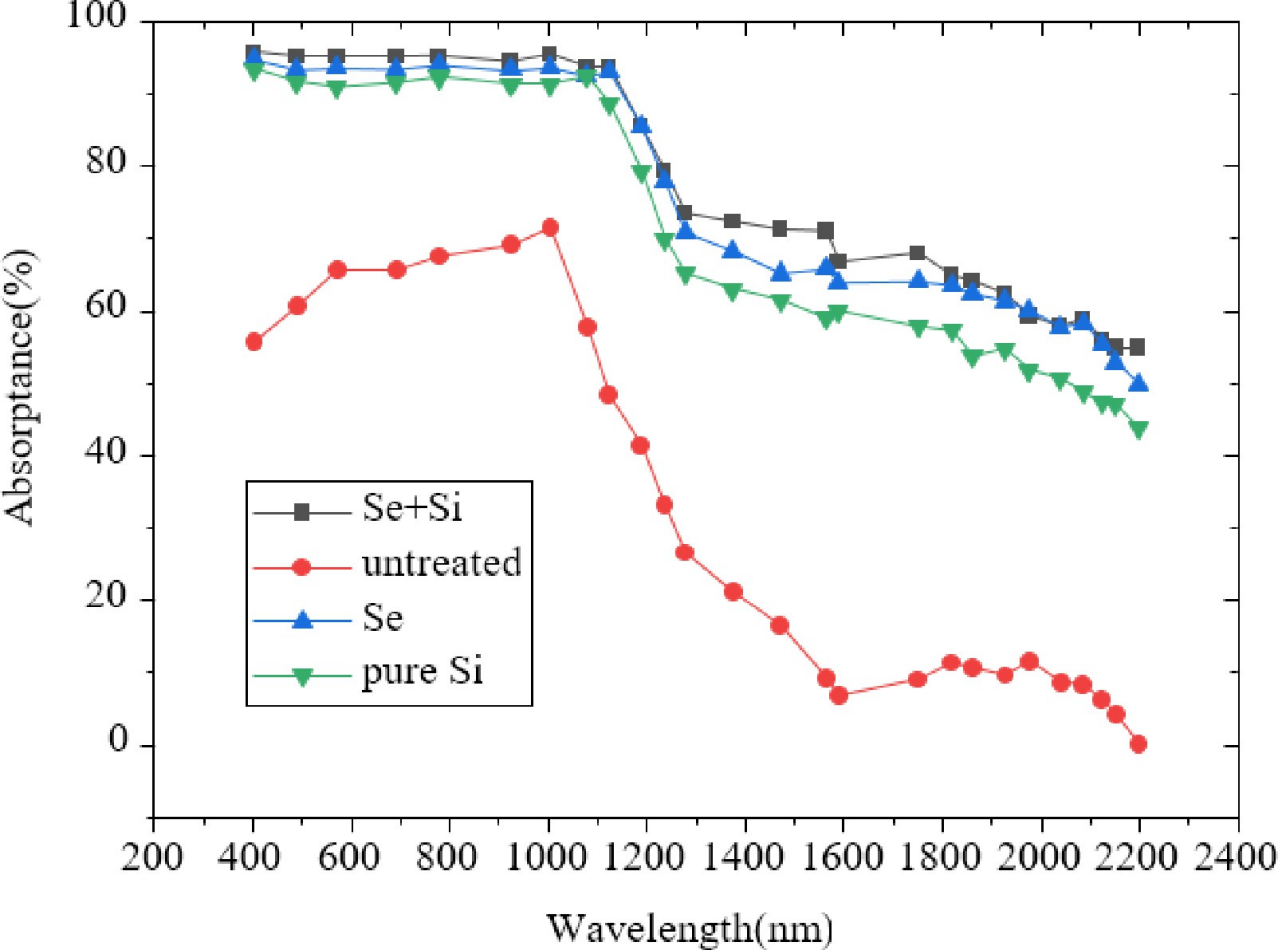
Comparison of absorption spectra of different film samples after FS treatment.

Fig 6 suggests that compared with the monocrystalline silicon sample without FS treatment, the black silicon prepared under the background gas of SF_6_ has greatly improved in 400-2200nm. Among them, in 400~1100nm, the optical absorption has increased from 60% to 95%, up about 35%. In the near-infrared band range of 1100~2200nm, the optical absorption has increased from about 10% to 90%, with an increase of 80%. Fig 7 illustrates the comparison of optical absorption of different film samples after FS treatment.

**Fig 8:**
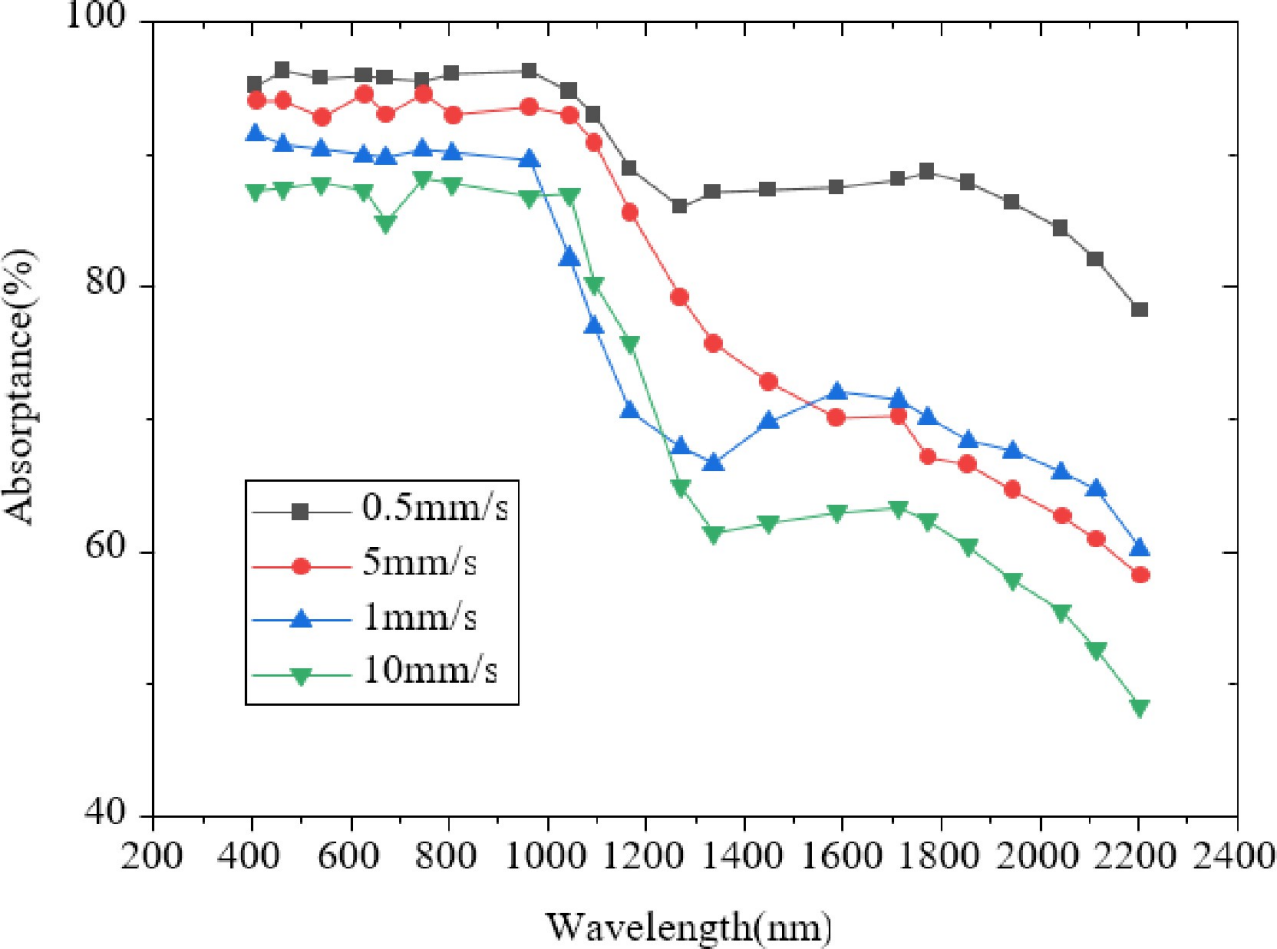
Comparison of absorption spectra of two-layer film samples at different scanning rates.

Fig 7 reveals that the optical absorption of black silicon treated by FS technology has improved. Among them, black silicon with a Se+Si two-layer film structure has the best optical absorption in the near-infrared range of 1100 nm~2200 nm. It shows that Se doping can improve the optical absorption of black silicon in the near-infrared range. Fig 8 presents the comparison results of absorption spectra of two-layer film samples under different scanning rates of FS.

**Fig 9:**
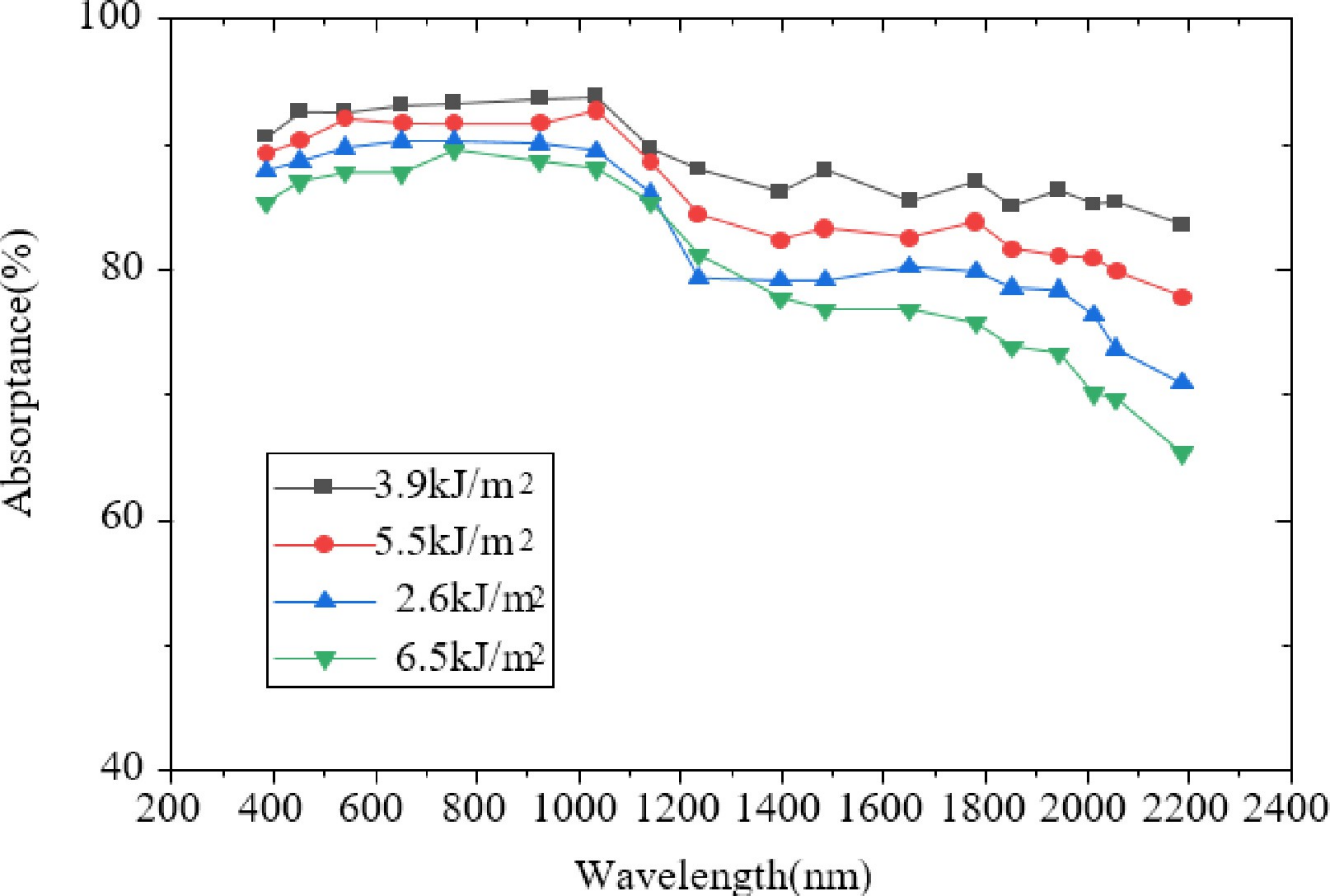
Comparison of absorption spectra of black silicon under different laser energy densities.

Fig 8 suggests that the absorbance of the absorption spectrum curve at different scanning rates in 400~2200nm shows a downward trend. The absorption rate is the highest and the effect is the best when the scanning rate is 0.5mm/s. The absorption rate is the lowest and the effect is the worst when the scanning rate is 10mm/s. It shows that the higher speed does not mean a better effect, while the most appropriate speed is needed to prepare black silicon samples. Besides, different scanning speeds represent the different number of FS pulses. Therefore, in order to prepare black silicon with the highest optical absorption, there must be enough laser pulses. Fig 9 displays the absorption spectrum results under different laser energy densities.

Fig 9 suggests that at the wavelength of <1100nm, the absorption rate of the etched two-layer film structure samples has little difference under different laser energy densities. However, in the band of >1100 nm, when the maximum laser energy density is 6.5kJ/m^2^, the overall absorption of the etched sample is the worst. When the laser energy density is 3.9kJ/m^2^, the absorption rate of the etched two-layer film structure sample is the best.

### 3.3 Discussion

To sum up, the different results of the above research have a profound correlation. Different background gases have the greatest impact on the properties of black silicon materials. The properties of black silicon treated by FS technology differ from the sample before treatment. The spectral absorption efficiency of two-layer thin film samples under different scanning rates of the machine is compared. When the scanning speed is 0.5mm/s, the black silicon material has the highest absorption rate and the best effect. Besides, in the etching process of an optical power splitter using FS technology, the selection of appropriate parameters, such as processing power and scanning speed, determines the final product effect. However, finding suitable process parameters by optimizing a single variable for control is difficult. Therefore, it is essential to comprehensively consider the processing power and scanning speed of FS technology in material processing, and conduct cross selection. This point is similar to the research results of Sima et al. (2018) [26]. They investigated the principle and application of FS 3D micro-nano machining in the application of the chip laboratory. Also, they introduced a hybrid technology that is expected to enhance the function of the chip laboratory equipment. It is found that different schemes can be integrated to realize more powerful microdevices, including on-chip laboratory devices. These devices can react, separate and synthesize biochemical materials with high efficiency, speed, and sensitivity, but low reagent consumption and waste. Similarly, Bai et al. (2020) used FS to directly induce a multi-level micro-column array on the surface of a thermally responsive shape memory polymer and successfully prepared a superhydrophobic memory surface [27]. This surface can change its surface morphology and wettability with heat. Ding et al. (2022) combined FS preparation and heat treatment, proposed a cactus palm layered structure, and clarified the optical absorption mechanism of multi-level structure based on numerical simulation [28]. This rapid preparation method will promote the large-scale industrial application of laser processing of photothermal conversion surfaces. It reveals that the preparation method proposed here has common points with similar research and also has its own advantages.

## 4. Summary and conclusions

Remote FS has a bright application prospect in ultra-fast, ultra-strong and ultra-precision machining. People favor the non-hot and cold processing properties of FS. They are applicable to the processing of high-precision and complex shape elements that other processing methods cannot realize. However, the control of the processing process and parameters is quite a complex problem, which will affect the final sample effect if people are not careful. Therefore, based on the principle and characteristic research of FS technology, this research proposes a scheme for preparing black silicon material through experiments and optimizes the experimental parameters by using the interaction between FS and silicon. Then, the scheme of using the FS for etching polymer optical splitter as a new technical means is proposed. The appropriate process parameters of laser etching photoresists are obtained while ensuring processing accuracy. By analyzing the characteristics of different forms of black silicon, the FS irradiation method is adopted to process and prepare black silicon materials. Through the vacuum system analysis of black silicon processing, the black silicon under different background gases is scanned by electron microscope in the experiment. The research has practical reference value for promoting the application of black silicon materials in preparing optical devices. Moreover, the experimental results of the research report show that black silicon technology has a pronounced auxiliary role in improving the efficiency of photovoltaic modules. Black silicon material has great application potential for improving the optical absorption capacity and cell efficiency of silicon wafers and achieving the best performance of photoelectric equipment. However, there are also some research defects, such as the problems that may occur in the specific packaging process of the optical power splitter, like coupling loss, signal transmission state in the path, and polarization sensitivity caused by the uneven structure. They need to be confirmed by further research, which is also the direction of future research work.

## Supporting information

S1 Data (RAR)
